# Conditions for Maintaining the Sustainable Development Level of EU Member States

**DOI:** 10.1007/s11205-017-1746-6

**Published:** 2017-09-05

**Authors:** Anna Bluszcz

**Affiliations:** Faculty of Mining and Geology, Silesian University of Technology, 44-100 Gliwice ul, Akademicka 2a, Gliwice, Poland

**Keywords:** Sustainable development, Synthetic indicators, Economic growth, Emission, Energy

## Abstract

For years we have been observing the exponential trend of the economic growth, energy consumption, mineral resources use and greenhouse gas emissions. The human population is exerting an increasing pressure on the environment, which in the highly industrialised regions has lost its natural ability for bio-capacity. The measurement of the member states’ progress in achieving the sustainable development is an integral part of the European Union strategy. The article deals with methods of measuring the level of sustainable development and presents diversification of the EU member states according to the synthetic indicators, such as: domestic material consumption, import dependency, risky external energy supply, diversity index, ecological footprint and total carbon intensity. These determinants affecting potential of the EU states to maintain the achieved level of development in future.

## Introduction

For years we have been observing the exponential trend of the economic growth, energy consumption and mineral resources use and greenhouse gas emissions. The human population is exerting an increasing pressure on the environment, which in the highly industrialised regions has lost its natural ability for bio-capacity. Maintaining the stable economic growth is the primary goal of all world economies, which means the long-term process of enhancement of goods and services in the given country. Classic determinants of the economic growth include: population size, availability of natural resources and efficiency of their use, assets and direct foreign investments, as well as expenses and the level of the budget deficit, globalisation, size and pace of the financial sector development. The economic growth means an increase in consumption of natural resources and increase in electricity consumption, which in turn affects the level of greenhouse gas emissions generated to the atmosphere.

The problem of studying these relationships requires a multidimensional analysis. The studies in particular concern the economies of the European Union countries; however, they also contain references to the phenomena on a global scale. On the basis of the conducted analyses it should be emphasized that the current global growth trends should be channelled to the sustainable levels, taking into account the needs of the future generations. Hence, the sustainable consumption and production are currently one of the most important challenges of the EU. Sustainable consumption and production was defined as: the holistic approach aimed at minimising the impact of the social production and consumption systems on the environment. The objective of sustainable production and consumption is to maximise the efficiency and effectiveness of products, services and investments, in order to meet the today’s needs of the society without compromising the ability of the future generations to meet their needs. This concept includes three pillars of sustainability: economy, society and environment. The social component is associated with the provision of generation and inter-generation justice and the consumer protection. The economic and environmental dimensions were described in the Kiev Declaration as the need for “eliminating the coupling between the economic growth and the environmental degradation in order to promote both the economic growth and the environmental protection”. Achieving this aim in the pan-European region was called the matter of fundamental importance (EEA 2007).

The development of humanity depends on the energy consumption, which next to food and air is one of the most important material needs of the man. The main factors influencing the increase in energy demand are: the growing population and economic growth of the world. In the past, the world population was small. In the middle of the seventeenth century the population exceeded 500 million. An increase to 1 billion occurred after almost 200 years, and the increase to 2 billion people was achieved in 1930. Therefore, an increase by 1 billion was achieved in just 100 years. In 1971 the population was already 3.7 billion, and during the last 45 years it has almost doubled, while in the last decades the dynamic increase in population occurred mainly in the non-OECD countries, especially in the underdeveloped countries of Africa, Asia and Latin America. Since 1971, there has been a rapid economic growth of the world—high in OECD countries and much lower in most non-OECD countries. Disparities are significant as the average gross domestic product (GDP) per capita in non-OECD countries was almost six times lower than the average GDP of the OECD countries in 1971 (respectively, GDP—OECD 13001 USD, and in non-OECD it was 2081 USD). While in 2011 the distance slightly decreased, as the GDP per capita income in OECD countries was about 5.4 times higher than in non-OECD countries and it was 30,545 USD, and in non-OECD countries it amounted to 5669 USD (Gilecki 2014).

With the increase in population there is a growth in consumption of goods and services, which increases the demand for the production of goods and services. Thus, for several generations we have been living in the world of unprecedented economic growth. In the twentieth century the world economy increased almost twenty times, at an average rate of 3% per year, which corresponds to doubling its size almost every 25 years. Because of this economic growth, people live longer and a better quality of life in comparison to their ancestors (Popkiewicz 2016).

The territorial expansion was an essential factor for the economic growth, which increased the resource base. Land resources are divided into renewable and non-renewable. The renewable resources can be sourced at a rate no faster than the rate of their re-growth without the loss for future generations. In the 1990s 20% of the land biomass production was used to meet the needs of the population in food, fuel and paper. In the first decade of this century this level increased to 25%, with this trend by the mid-century we will have used more than half of the whole land biomass.

According to the Organisation of the United Nations for Food and Agriculture, 38% of the Earth’s ice-free area was converted into agricultural lands (including 12% for crops, 26% for pastures). Crops already represent 70% of the grasslands, 50% of the savannas and once occupied by forests, and 27% of tropical areas. From 1985 to 2005 there was a significant expansion of tropical areas, what has a huge impact on biodiversity, emissions and soil conditions (Foley 2011).

The primary source of information about the energy resources are the Survey of Energy Resources carried out by the World Energy Council. These surveys use two basic concepts, namely:

- Geological resources (*resources*—*proved amount in place*) are the total amount of the documented reserves of energy resources.
- Operative resources (*proved recoverable reserves*) are a part of the resources, which can be extracted in the current technical and economic conditions.

From the review of the energy resources conducted in 2013 it results that despite the growing extraction, the size of the operative fossil resources is growing. The comparison of the data presented in the reviews of 1974 and 2013 [BT] (evaluation published at the end of 2011) indicates the following increase of the operative resources of: coal by 145% (from the level of 476 [BT] in 1974 to the level of 690.5 [BT] in 2013); oil by 204% (from the level of 89.7 [BT] to the level of 182.8 [BT]); natural gas by 324% (from the level of 64.8 [BT] to the level of 209.7 [BT]). A significant increase in resources, especially of hydrocarbon fuels, is the result of new discoveries, technological progress in the search for new resources, exploitation of submarine resources, etc. Unfortunately, this is related to a considerable increase of the fuel extraction costs. The distribution of fossil fuel resources is very uneven in the globe. The largest deposits of coal can be found in the USA, Russia, China and India, and of lignite—Germany, Australia, the USA and China. Rich oil reserves can be found in the Arab countries, especially Saudi Arabia, Iran, Iraq, Kuwait, United Arab Emirates, Libya, as well as Venezuela and the Russian Federation. The largest natural gas reserves are located in the Russian Federation, Iran, Turkmenistan, Qatar, Saudi Arabia, the USA, the United Arab Emirates and Venezuela. The inspections of the energy resources also contain information on the rich resources of bitumen, i.e., the shale and bitumen sands, and heavy oil. It is estimated that the bitumen resources contain about 1.5 trillion of oil Mg. Particularly important are the resources of tar sands in Canada and the heavy oil in Venezuela. Tar sands in Canada and heavy oil in Venezuela are already operated on an industrial scale, and the costs of obtaining oil from these bitumen are similar to the oil prices in the international trade (Gilecki 2014).

## Materials and Methods

Sustainable development is a general concept characterized by numerous definitions and many possible interpretations of its meaning. The two most popular approaches will be included in the article. The first approach means development which meets the needs of current generations without compromising the ability of future generations to meet their needs and aspirations (WCED 1987). The second approach means development which improves the quality of human life while living within the carrying capacity of supporting ecosystems. This approach is concerned with quality-of life issues and maintenance of the ecological integrity of natural systems (IUCN 1991).

The sustainable development is a complex and interdisciplinary issue, covering economic, social and environmental aspects, thus there are many concepts of its measurement, since there is no a single universal methodological tool to measure it. Most often aggregated synthetic gauges describing a social, economic and environmental spheres at the same time are used to evaluate the level of the sustainable development, and they should be characterized by the following primary principles: (Button 2002; Repetti and Desthieux 2006). The indicator system should embody the social, economic, ecological, environmental spheres and objectively reflect the scientific consensus on sustainable development. The meanings of the indicators should be independent to avoid overlap and autocorrelation and measurable using appropriate quantification techniques. The difficulty of collecting and quantifying the data and indicators must be low enough to permit practical use of the indicator. Indicators should be sensitive to temporal, spatial, or structural changes in the system to reflect the changes of the social, economic and environmental development and capture long-term processes.

The previous research in the scope of methods of measurement of the level of sustainable development of the countries are mainly focused on the problem of selection and choice of optimal set of indexes and examining their changes in time. This article uses available information in the scope of assessment of the EU states as per the environmental aspects of the sustainable environment indicating the need of a broader assessment, it means including the potential of the EU member states to maintain the achieved development level in a long time prospect, the elaboration of the potential measurement method, first of all, required: identification of the main determinants of the potential to maintain the determined level of the development in a long period of time until their examination of their influence of that potential. Based on the models prepared by international institutions such as: UN Commission on Sustainable Development (CSD), the UN Department for Policy Coordination and Sustainable Development (DPCSD), UNSTAT and the Scientific Committee on Problems of the Environment (SCOPE) (MSD 2008) the main environmental determinants of sustainable development included, among others: consumption of natural resources, energy consumption, the level of natural environment use and its pollution through the emission of greenhouse gases. The article analyses the dependency between the identified determinants, which influence the ability of the member states to maintain, in a long time prospect, the determined level of the development hereinafter referred to briefly as the potential of the sustainable development—PSD.

The identified determinants of the sustainable development should be of a measurable character and thus the following set of adequate indexes was chosen for the analysis. The consumption of natural resources was characterized by domestic material consumption (DMC); the energy consumption was expressed by means of the level of energy consumption (EC) and the consumption level in relation to the gross domestic product (EC/GDP) in order to obtain a unit comparable between the states. A critical factor affecting the potential of a state to maintain the achieved development level is securing energy supplies, thus the following indexes were adopted for the analysis: energy efficiency (EE), import dependency (ID), risky external energy supply (REES) and the Shannon–Wiener diversity index (H).

The level of the natural environment use was characterized by means of gauge Ecological Footprint whereas the level of the atmosphere pollution was described with gauge Carbon Footprint. All indexes selected for the analysis have a significant influence on the EU member states’ potential to sustain the achieved development level, which was presented in Fig. 6.

## Results

### The Analysis of Influence of DMC and RP on the States’ Potential to Sustain the Achieved Development Level

Domestic material consumption (DMC) includes all materials directly used in economic processes for needs of national economies. DMC is the sum of materials obtained in the country territory and from import reduced by the exported materials. The index of domestic material consumption (DMC) is based on Economy-wide Material Flow Accounts, it is coherent compilation of total material amount flowing into the national economies, change of material reserves level in economy and material outflows to other economies or to the environment. For data comparability DMC level per capita was adopted in the analysis.

The member states are significantly different as regards DMC index. The lowest level in 2015 was achieved by Italy 6.9 tonnes per capita, Spain 8.3 tonnes per capita, whereas the highest level was achieved by Finland 30.5 tonnes per capita and Estonia 27 tonnes per capita. Moreover, there occur differences in the consumption structure in conformity with main categories, such as: non-metallic minerals, biomass, fossil energy materials and metal ores.

The DMC of the aggregated EU-28 economy is dominated by non-metallic minerals making up nearly half of the DMC in 2013 (6.2 tonnes per capita). With 3.4 and 3.1 tonnes per capita respectively, biomass and fossil energy materials each make up approximately one fourth of DMC. Metal ores constitute the smallest of the main categories with 0.5 tonnes per capita. The level of DMC differs greatly among EU Member States, ranging from 8.4 tonnes per capita in Spain to 34.5 tonnes per capita in Finland in 2013. The composition of DMC in each EU Member State is influenced by domestic extraction and by natural endowments with material resources, and the latter may form an important structural element of each economy. The consumption of non-metallic minerals was lowest in the Netherlands (2.2 tonnes per capita) and highest in Finland (19 tonnes per capita). Non-metallic minerals constitute a significant part of DMC in several other EU Member States, notably Romania (16.2 tonnes per capita), Estonia (12.2 tonnes per capita), Ireland and Austria (11.3 and 12.5 tonnes per capita respectively). Consumption of biomass was highest in Latvia (10.7 tonnes per capita), Ireland (9.1 tonnes per capita), Lithuania (7.1 tonnes per capita), Finland (6.9 tonnes per capita) and Sweden (5.6 tonnes per capita). In Ireland, fodder crops and grazed biomass made up the biggest share of this category, while in the other EU Member States with high values forestry played a major role in the economy. Consumption of biomass was lowest in Malta (1.4 tonnes per capita). EU Member States with substantial amounts of fossil fuel consumption included Estonia (14.7 tonnes per capita, due to oil shale), Greece (6.2 tonnes per capita), the Czech Republic (5.9 tonnes per capita), Germany (5.3 tonnes per capita, due to lignite) and Bulgaria (5.0 tonnes per capita), Portugal and Latvia reported the lowest consumption among EU Member States for fossil energy materials, each with 1.4 tonnes per capita. Finally, consumption of metal ores was highest in Sweden (5.7 tonnes per capita), Bulgaria (4.0 tonnes per capita) and Finland (4.0 tonnes per capita) because of their metal mining activities. The lowest values among EU Member States were reported in Estonia and Lithuania (Energy 2015).

The key indicator of the material use efficiency in a specific economy is the resource productivity index describing what level of economic value the economy of this country was able to produce using one kilogramme of the material [euro/kg]. This index is the leading one in the theme of sustainable production and consumption. The level of this index in the EU states in 2015 was the highest for the following countries: UK (4.48); Luxembourg (4.2), Italy (3.9), the Netherlands (3.6) and Germany (2.3), whereas the lowest level was achieved by: Bulgaria (0.3), Romania (0.35), Latvia (0.56), Estonia (0.57) and Poland (0.67) on the grounds of the presented data it can be stated that the highest DMC levels the lowest potential of the country to sustain the achieved level of sustainable development in future, whereas the higher resource productivity RP level the higher the potential of the country.

### The Analysis of Influence of Energy Consumption on the Potential of Countries to Sustain the Achieved Development Level

Among 28 EU member states in 24 countries there was an increase in electricity production. The highest increase was observed in Luxembourg 123%, Cyprus and Malta above 110%, Spain, Portugal and Ireland above 80%. The decrease was observed in: Estonia, Romania, Latvia and Lithuania (Eurostat 2013).

Total production of primary energy for the EU-28 was 789.7 million tonnes of oil equivalent (TOE) in 2013. The EU-28’s major primary energy producers were France (17.1%), Germany (15.3%) the United Kingdom (13.9%) followed by Poland (8.9%) and the Netherlands (8.8%) (Energy 2015).

**Fig. 1:**
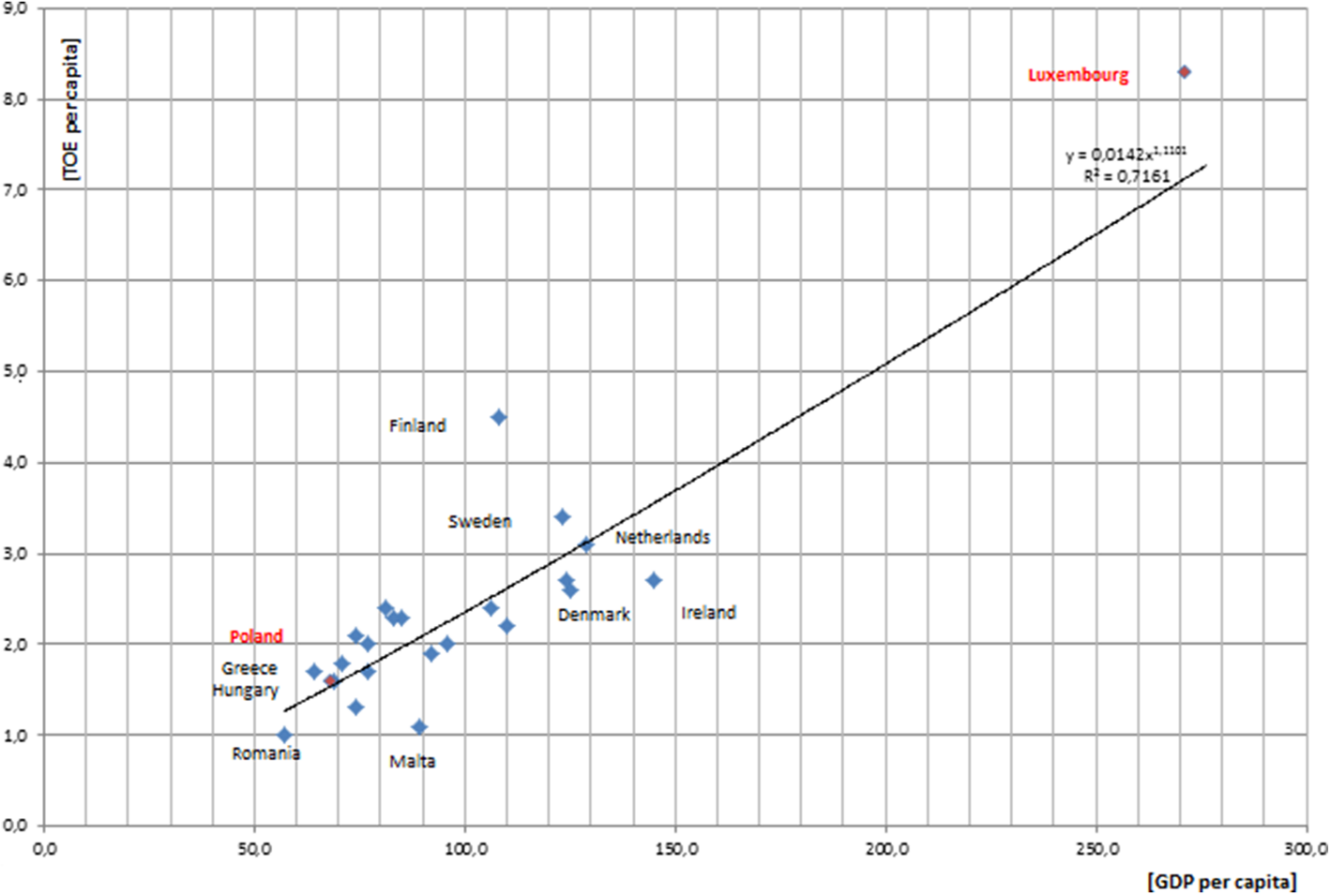
Energy consumption in relation to the gross domestic product. *Source*: Own elaboration based on data from: http://www.eea.europa.eu/data-and-maps/figures/final-energy-consumption-million-toe-3, http://ec.europa.eu/eurostat/statistics-explained/index.php/File:Volume_indices_per_capita,_2012-2015_(EU-28%3D100)vJune.png

The level of electricity consumption in the EU countries is diverse from 1 TOE/capita in Romania to 8.3 TOE/capita in Luxembourg as was shown in Fig. 1.

The energy consumption per capita in the EU countries is dependent on the GDP per capita because new member states with much lower income also have a lower energy consumption, i.e., an average citizen of Luxembourg or Norway consumes about three times more energy than an average citizen of Portugal, Greece or Poland.

Maintaining a constant economic growth is dependent on the stable access to energy resources. Ensuring an uninterrupted source of supply is a prerequisite for ensuring the energy security of each country.

According to the International Energy Agency (IEA) energy security is define as the uninterrupted availability of energy sources at an affordable price. A standard definition of security of supply is a flow of energy supply to meet demand in a manner and at a price level that does not disrupt the course of the economy in an environmental sustainable manner (IEA 2014).

Energy security ought to be comprised of five dimensions related to *availability*, *affordability*, *technology development*, *sustainability* and *regulation* (Sovacool and Mukherjee 2011).

The EU countries belong to the countries poor in the oil and gas reserves; hence their high consumption depends on the import of these fuels.

Import dependency shows the extent to which a country relies upon imports in order to meet its energy needs. It is calculated using the following formula (EEMS 2013):

1 $$ID_{j} = \frac{{M_{j} - X_{j} }}{{GIC_{j} + Bunk_{j} }}$$

X—export, M—import, J—energy product, GIC—gross inland consumption, Bunk—Consumption of International Bunkers, Unit = %.

The average rate of energy dependence for the EU countries in terms of all products amounted to 50.2% in 2004 and still has an upward trend, as in 2013 it already amounted to 53.2%. The highest level of dependence is in terms of the import of the total petroleum products of 79.7% in 2004 and 87.4% in 2013. Dependence in relation to the supply of natural gas also increased, resulting in 2004 in the level of 53.6% and in 2013 65.3%. The EU countries are also dependent in terms of the import of solid products on average in 2004 at the level of 38.2%, while in 2013 at the level of 44.4%. More details in (Energy 2015).

Among the member states there is a considerable variation in terms of energy dependence. The highest dependence in terms of oil, gas and coal in 2013 was shown by Malta (104.1%), Luxembourg (96.9%), Cyprus (96.4), Ireland (89%), Lithuania (78.3%), Belgium (77.5%) and Portugal (73.5%). In contrast, most independent of energy are: Estonia (1.9%), Denmark (12.3%), Romania (18.6%), Poland (25.8%), Netherlands (26%), Czech Republic (27.9%) and Sweden (31.6%). The broader reference to the issues of energy consumption and the level of dependence of the EU countries in terms of the energy resources can be found in the paper (Bluszcz 2016a, b).

The energy dependence is also connected with the risk of supply, which is measured by the Risky External Energy Supply (REES) index, calculated independently for each energy media separately, i.e., for gas, oil and coal, according to the following formula (Cog and Paltseva 2009):

2 $$REES_{a}^{f} = \left[ {\sum\limits_{i} {\left( {\frac{{NPI_{ai}^{f} }}{{NPI_{a}^{f} }}} \right)^{2} F_{ia}^{f} r_{i} d_{ia} } } \right] \cdot NID_{a}^{f} \cdot SF_{a}^{f}$$

where $NPI_{ai}^{f}$—the net positive imports of fuel *f* from country *i* to country *a*, $NPI_{a}^{f}$—the sum of net positive imports over all suppliers of country *a*, $F_{ia}^{f}$—the fungibility of imports of fuel *f* from country *i* to country *a, r*_*i*_—the political risk index of the supplier country, *d*_*ia*_—is a measure of a distance between countries *i* and *a*, $NID_{a}^{f}$—the net import dependency of country *a* from fuel *f*, $SF_{a}^{f}$—a share of fuel *f* in country *a.*

Higher values of REES index correspond to higher risk. According to Cog’s et all calculations carried out based on the 2006 data for the EU member states, the highest level of the risk indicator for the oil characterised Hungary (18.3), Slovakia (10.8), Lithuania (10.2) and Bulgaria (10.4). The risk indicator for the supplies of gas was the highest for Slovakia (39.4), Hungary (33.6), Latvia (21), Lithuania (20.1), Bulgaria (17.5) and Austria (16.7). Risk indicators for the supply of coal are the lowest in the group of energy resources for all member states, and the highest level was noted for Romania (6.4), Denmark (5), Ireland (4.7) and Slovenia (5.4).

Another indicator determining the level of energy security of the countries in terms of the degree of diversification of energy supply is the Shannon–Wiener diversity index (H). In mathematical terms the function is denoted as follows (Stirling 1994; EC 2010; Kaliski and Staśko 2007; Kruyt et al. 2009):

3 $$H = - \sum\limits_{i = 1}^{n} {p_{i} \log (p_{i} } )$$

where *p*_*i*_—share of the i-th energy carrier, n—number of energy carriers.

**Fig. 2:**
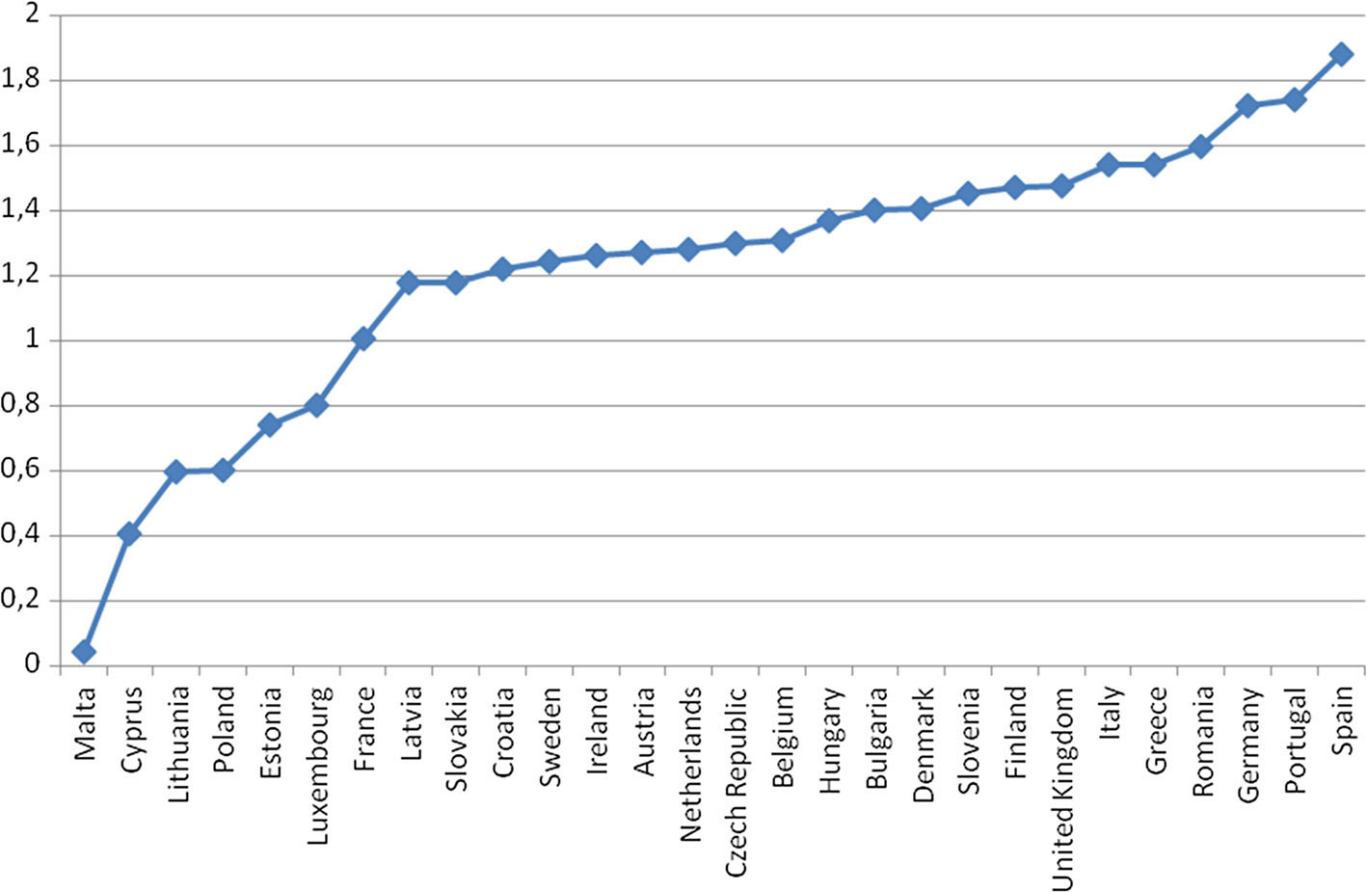
Calculation of the diversification H index. *Source*: Own elaboration basis on EC—European Commission: EU Energy, Transport and GHG Emissions. Trends to 2050 Reference scenario 2013 Publication Office of the European Commission 2013

The higher the value of H the greater the diversity. Figure 2 presented the calculations of the H indicator for the EU countries.

The EU countries are characterised by the diverse level of supply diversification of energy sources measured by the H index, the lowest diversification level can be observed for Malta and Cyprus, where over 90% of gross electricity generation in GWhe comes from oil (including refinery gas). Poland and Estonia also belong to the countries with a low degree of diversification due to the high share of solid fuels in gross electricity generation in GWhe. Luxembourg and Lithuania are characterised by the highest share of gas in gross electricity generation in GWhe. The highest level of the diversification index in the EU is in Spain, Portugal and Germany.

Based on the presented analysis it can be stated that the higher the energy consumption level the lower the level of the country’s potential to sustain the achieved development level. The higher the level of the energy dependency the lower the potential level. The higher the level of Risky External Energy Supply (REES) index the lower the potential level and the higher the level of H index the higher the potential level, which was presented in Fig. 6.

### The Analysis of the Influence of the Natural Environment Use and Its Pollution on the Potential of Countries to Sustain the Achieved Development Level

The most popular gauge of the natural environment use by human activity is Ecological Footprint. EF identifies six categories of areas measured literally with the unit areas (so-called global hectares), i.e., agricultural and farm use of the land, forests, fishing areas, urban areas, green areas absorbing carbon dioxide. The ecological footprint shows, above all, to what extent the areas with high industrialisation rely in natural resources located in other parts of the world. For each area we calculate the rate, expressed in area units, in global hectares per person (Gha/person). EF calculations are regularly published in the reports of WWF Living Planet 2012 (LPR 2012).

**Fig. 3:**
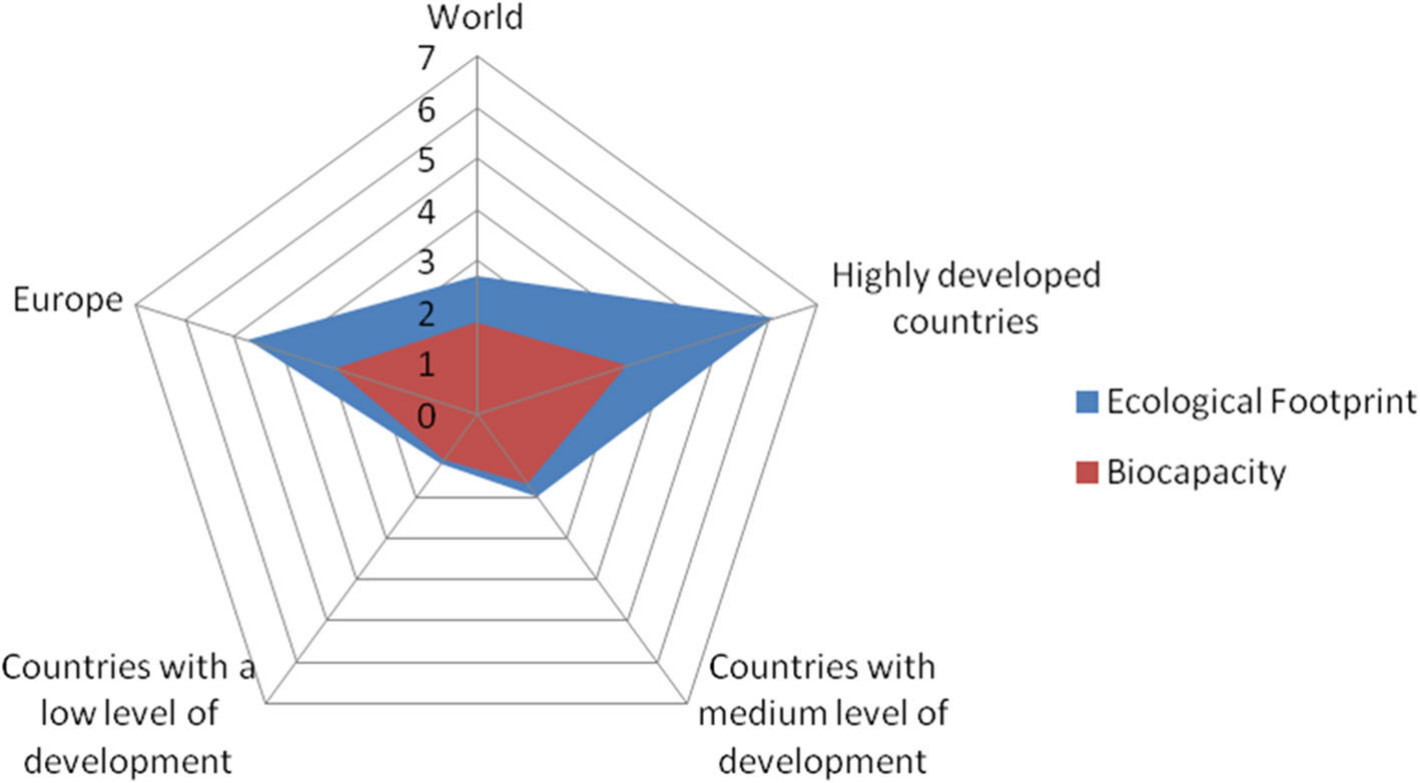
The relation of the ecological footprint to the bio-capacity. *Source*: Own elaboration based on Global Footprint Network

Based on the data presented on the Global Footprint Network websites, Fig. 3 presents the exemplary estimations of the ecological footprint for the world with the division into the highly, medium and low developed countries, including Europe.

According to the data, the average ecological footprint 2.7 Gha/person for the world exceeds the average biological capacity of 1.8 Gha/person, hence the ecological deficit of an average 0.9 Gha/person. Human population in the highly developed countries, where 15% of the world population inhabits, shows the highest level of the ecological footprint of 6.1 Gha/person in relation to biocapacity 3.1 Gha/person generating the ecological deficit in the amount of 3.0 Gha/person. In countries with a medium level of development, with 65% of human population, the deficit is 0.2 Gha/person. While in the countries with the lowest level of development, the ecological deficit is 0.1 Gha/person. Considering the presented data, this means that 75% of the ecological deficit generated by the human population is created in highly developed countries.

In Europe only four countries have the ecological reserve, that means that they use less resources than their biological capacity, these are: Finland (6.3 Gha/person), Sweden (3.9 Gha/person), Latvia (1.4 Gha/person) and Estonia (1.1 Gha/person). Countries with the highest ecological deficit include: Belgium (6.7 Gha/person); the Netherlands (5.2 Gha/person); Spain, Italy and Greece with the deficit at the same level of 3.8 Gha/person, Great Britain (3.6 Gha/person), Denmark (3.4 Gha/person), Germany and Portugal (3.2 Gha/person). More in Bluszcz (2016a).

The existing disparities between the individual EU countries in the consumption of natural resources per capita, the electricity consumption and in efficiency their use raise the need to work towards their sustainable use.

The analysis of Ecological Footprint index indicates that its increase affects the decrease of the country potential to maintain the achieved sustained development over a long period of time, because nature’s abilities to regenerate are limited.

**Fig. 4:**
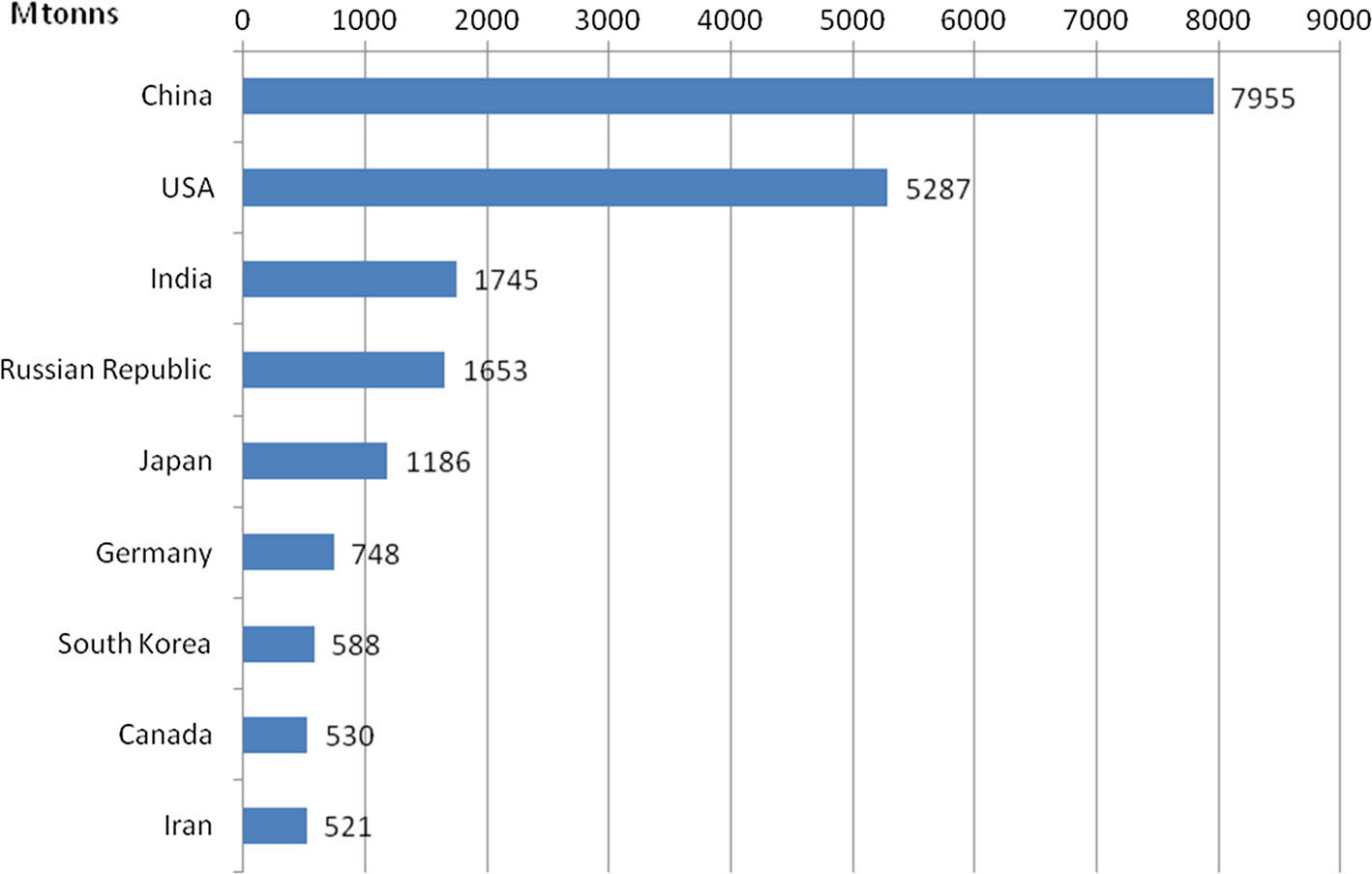
Countries with the highest emission of CO_2_ in 2011. *Source*: Gilecki R.: The energy sector of the world and Poland. The beginnings, development, current status. Warsaw 2014

**Fig. 5:**
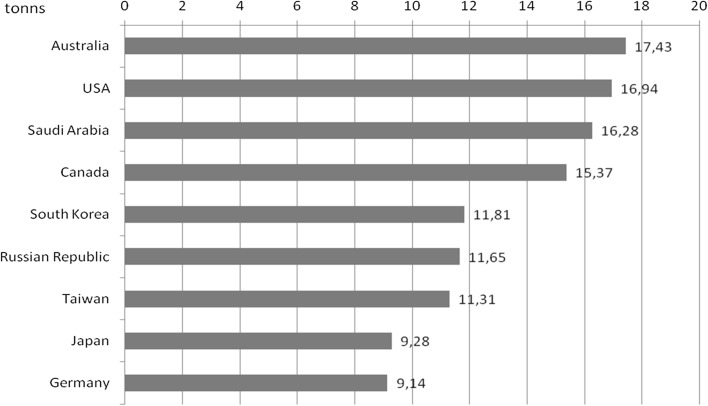
Countries with the highest emission of CO_2_ per capita in 2011. *Source*: Gilecki R.: The energy sector of the world and Poland. The beginnings, development, current status. Warsaw 2014

The use of the natural environment to satisfy human needs is linked with phenomena of global climate warming, because people, through their consumption and production decisions emit GHGs. Carbon dioxide is especially important, accounting for around three-quarters of the human-generated global warming effect; other relevant GHGs include methane, nitrous oxide, and hydrofluorocarbons (HFCs). These flows accumulate into stocks of GHGs in the atmosphere. It is overall stocks of GHGs that matter, and not their place of origin. The rate at which stock accumulation occurs depends on the “carbon cycle,” including the earth’s absorptive capabilities and other feedback effects. The stock of GHGs in the atmosphere traps heat and results in global warming: how much depends on “climate sensitivity.” The process of global warming results in climate change, affects people, species, and plants in a variety of complex ways, most notably via water in some shape or form: storms, floods, droughts, sea-level rise. These changes will potentially transform the physical and human geography of the planet, affecting where and how we live our lives (Stern 2008). The level of global emission is considerably varied, and is presented in Figs. 4 and 5.

In the emission balance in the European Union countries in 2012 the greatest share goes to the following sectors: energy 57.9%, transport 22%, agriculture 10%, industrial processes 6,9%, waste 3%, solvent and other product use 0.2%. In relation to 1990, the highest growth by 7% was noted in the transport sector, the drop by 1.2% in the sector of industrial processes, and the drop by 4.3% in the energy sector (IEA CO_2_ 2014).

According to the data of the Netherlands Environmental Assessment Agency report, the share of the member states in the global emission of greenhouse gases in 2013 was 10.5%, while it should be noted that the level of emission dropped by 8.4% in relation to 1990. Currently, China is the largest emitter, whose level of emission amounted to 29.2% in 2013, and drastically increased by 18.2% in comparison to 1990. The United States account for 15% of the global emission and their share dropped by 7% in relation to 1990. The Russian Federation generates 5.1% of the global emission (decline by 5.5%), Japan 4% (decline by 1.3%), India 5.9% (increase by 2.8%), international transport—an unchanged level of 3.1% (NEAA 2014).

The size of total emissions is not the only measurable indicator of the emission of economies, because in regard to the analysis according to the emission per capita, the situation of countries undergoes different trends. This means, that countries with the highest level of emission at the global scale per capita are the US, Canada Australia, achieving the level of almost 20 tons CO_2_ per capita in 2004 (Stern 2008) while in 2011 the levels of emission were, respectively, 16, 15 and 17 tons CO_2_ per capita (Gilecki 2014). In the European Union countries the highest level of emission in 2012 characterised Luxembourg (20.7 tons CO_2_ per capita), Estonia (12.9), the Czech Republic (10.6), Iceland (10.4), Germany (10), the Netherlands (9.9), Finland (9.4), Belgium (9.1) and Norway (8.9). Countries with the lowest level of emission of CO_2_ per capita were: Latvia (3.6), Romania (4.2); Croatia (4.5), Hungary (4.6), Lithuania (4.7) (Eurostat 2012). More in Kijewska and Bluszcz (2016), Brodny and Tutak (2016a, b).

The research results of the dependency between carbon dioxide emissions and economic growth according to Kuan-Min Wang, which were conducted on data from 138 countries in the years of 1971–2000 are as follows:

- The long-run relationship between global carbon dioxide emissions and GDP is stable, with 32.6% of sampled countries showing cross-coupling of the two (with an elasticity value over 1), 47.1% reporting relative-decoupling (with the elasticity value between 0 and 1) and 20.3% seeing absolute-decoupling (with the elasticity value under 0).
- The quantile regression shows that the long-run elasticity declines along with the rise of carbon dioxide emission quantile regressions mostly support the feedback relationship between carbon dioxide emissions growth and economic growth.
- The relationship between these two is steady and feedback in the case of high quantiles.

Therefore, the first propriety to combat global warming is to focus on the countries with the high economic growth and high carbon dioxide emissions growth (Wang 2013).

**Fig. 6:**
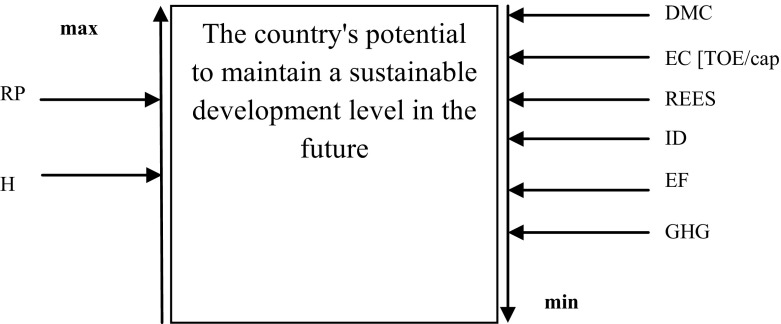
Influence of selected indexes on PSD potential level. *Source*: Own elaboration

On the grounds of the conducted analysis it can be stated that the higher the level of economy emission the lower the level of the country’s potential to maintain the sustainable development in future, which was presented in Fig. 6.

The analysis presented in this paper demonstrate how much the popularization of initiatives and activities of the member states for changes, among others, in the following scopes is needed: the de-carbonisation initiatives as well as the increase of efficiency of the energy use, growth of the renewable fuels use and the rational use of the non-renewable resources for maintaining the ability to satisfy the needs of temporary and future generations.

## Conclusions

Permanent economic growth and population growth exert considerable pressure on the environment. The impact of this is not equal on a global scale and in Europe.

The production of electricity, the supply of gas, fuel and water, mining, production of metals and industrial minerals, transport and agriculture are the sectors exerting the largest pressure on the environment. Forecasts of the consumption of natural resources have the growing trend up to 2020, which means the urgent need for sustainable development actions. Maintaining the current level of consumption of highly developed countries at the current rate of the population growth already in the near future can lead to the exhaustion and depletion of non-renewable natural resources, and the environment in the areas of countries with the high level of the ecological footprint with the natural opportunity to rebuild (Bruyninckx 2015).

The economic status quo cannot be maintained long into the future. The economy must be transformed so that it can be sustained over the long run. It must follow three precepts:

- Limit the use of all resources to rates that ultimately result in levels of waste that can be absorbed by the ecosystem,
- Exploit renewable resources at rates that do not exceed the ability of the ecosystem to regenerate the resources.
- Deplete non-renewable resources at rates that, as far as possible, do not exceed the rate of development of renewable substitutes (Daly 2005).

The first propriety to combat global warming is to focus on the countries with the high economic growth and high carbon dioxide emissions growth.

By taking these challenges, the European Union established the leading objectives to be achieved in the period of 2020–2030. They mainly concern the reduction of GHG emissions by 40% by 2030 compared to 1990, increasing the share of renewable energy to at least 27% of energy consumed in the EU in 2030, and improved energy efficiency by at least 27% in 2030 (EPPSA). Evans analysis indicates that the wind power is the most sustainable renewable energy technology followed by hydropower, photovoltaic and then geothermal ones. Wind power was identified with the largest relative greenhouse gas emissions, the least water consumption demands and with the most favourable social impacts compared to other technologies, but requires larger land and has high relative capital costs (Evans et al. 2009).

Another aspect is the sustainable use of resources in the long term, which requires taking into account their availability, ensuring security of supply and protection of ecosystems. At the same time, it is important to maintain the ability of the environment to absorb the emission and pollutants. The improvement of efficiency, adoption of innovative technical and managerial solutions and a better monitoring and control of the environment are required to improve sustainability in production. Measures to ensure the safety of energy resources supply in the dynamically changing geopolitical situation require active search for the reliable and diversified supply of energy resources and changes in the internal energy market in the EU towards their integration.
